# Asymmetric polyhedron structured NiSe_2_@MoSe_2_ device for use as a supercapacitor

**DOI:** 10.1039/d0na01047b

**Published:** 2021-06-02

**Authors:** M. Sangeetha Vidhya, R. Yuvakkumar, G. Ravi, B. Saravanakumar, Dhayalan Velauthapillai

**Affiliations:** Department of Physics, Alagappa University Karaikudi 630 003 Tamil Nadu India yuvakkumarr@alagappauniversity.ac.in; SARP, LARPM, Central Institute of Plastic Engineering and Technology (CIPET) Bhubaneswar 751024 India; Faculty of Engineering and Science, Western Norway University of Applied Sciences Bergen 5063 Norway

## Abstract

The polyhedron-structured NiSe_2_@MoSe_2_ (NMS) were magnificently produced through hydrothermal method, which makes the active electrode very effective in accessing the redox active sites in the charge/discharge process in supercapacitor applications. Systematic studies of the structural and morphological features of the unique polyhedron NMS were performed. Laser Raman spectra reported the vibrational and rotational modes of NMS. NMS exhibited a maximum 1000 F g^−1^ (138.8 mA h g^−1^) specific capacitance at 1 A g^−1^ and attained a capacity retention of 77.67% for 5000 stable cycles at 10 A g^−1^ using 1 M KOH as an electrolyte. The well assembled asymmetric supercapacitor NMS hydrolyzed for 18 h//AC demonstrated an outstanding electrochemical performance with an improved 305 F g^−1^ specific capacitance attained at 1 A g^−1^ with a good cycle life of 87.35% and a capacity retention of over 5000 cycles. Herein, the combined contribution of both the Ni and Mo ions offers a richer redox chemistry, which is beneficial to a higher electrochemical activity. The experimental results indicate that the NMS electrode could be used as a high-performance electrode.

## Abstract 2

Approaching into the part of redox active sites in charge/discharge material is extremely noteworthy for the progress of high performance of the polyhedron structured NiSe_2_@MoSe_2_ (NMS) hydrolyzed for 18 h as active electrode material.

## Graphical abstract

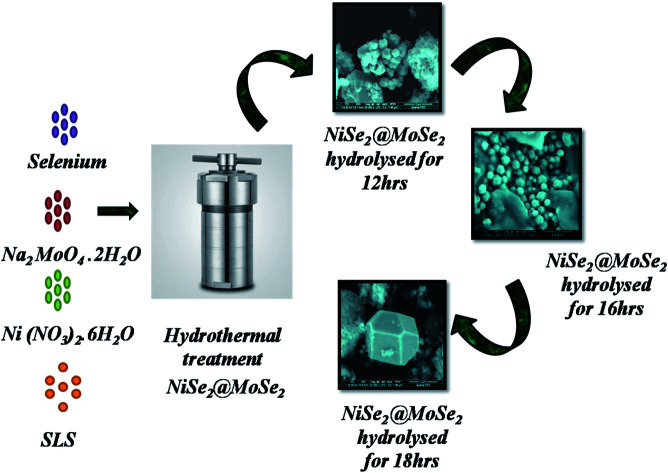

## 1. Introduction

The fall of fossil fuels has led many researchers to show their interest in the development of energy harvesting and storage devices.^1,2^ Significantly, supercapacitors have attracted much attention compared to various energy storage devices due to their power density,^3,4^ long service life^5,6^ and drastically lower cost of critical components.^7,8^ Compared to various energy storage devices, electrochemical capacitors, also called supercapacitors, have attracted attention because of their power density,^3,4^ long term service life^5,6^ and the low cost of the significant importantly components.^7,8^ Furthermore, abundant studies have concentrated on enhancing the performance of the electrode material. According to the charge-storage, the asymmetric supercapacitor strongly relies on the material structure and hence an active electrode material was designed and fabricated.^9,10^ Moreover, the active element is the key factor effecting the progress of the asymmetric device. In general, focus has been mainly concentrated on the performance of the active electrode material for supercapacitor applications, the charge storage mechanism of the electric double layer capacitor through the adsorption/desorption ion at the electrode surface, and the fast faradaic reaction for pseudocapacitors (PC).^11^ Hybrid capacitors based on carbon materials, conducting polymers, transition metal oxides/chalcogenides are generally used in supercapacitors. The poor electrode presentation occurs due to the sluggish ionic transport kinetics. Subsequently, there is an urgent need to develop short ionic diffusion distances, high electrical conductivity and good electrochemical stabilization.^12^ Recently, transition metal selenides have been regarded as promising candidates with a good conductivity and electrochemical activity.^13^ In particular, metal selenide shows superior electrochemical properties with regards to the material architecture, by enhancing the large surface area enabling and accelerating the redox reaction.^14^ Furthermore, Se belongs to the same group as the O element and shares similar physical and chemical properties.^15,16^ The material Se has good metallic features; therefore, the corresponding transition metal composites exhibit a good electronic conductivity compared to the transition metal oxides (TMOs) which is vital for electrochemical applications.^17^ The presence of the active site on the layered MoSe_2_ results in an enhanced electrochemical and electronic conductivity.^18^ Moreover, transition metal doping has become an important technique for enhancing the electrochemical properties.^19^ Over the last two decades, Ni based transition metal selenides have emerged as promising active materials for supercapacitor applications. Here, NiSe_2_ offers higher charge storage capability, while MoSe_2_ is used to improve electrical conductivity rather than providing higher Faraday redox reactions during the charge/discharge process. Hence, in the present work, the polyhedron structured NiSe_2_@MoSe_2_ (NMS) was synthesized using the hydrothermal route and improves the performance of the electrochemical capacitor.

## 2. Experimental

**Fig. 1:**
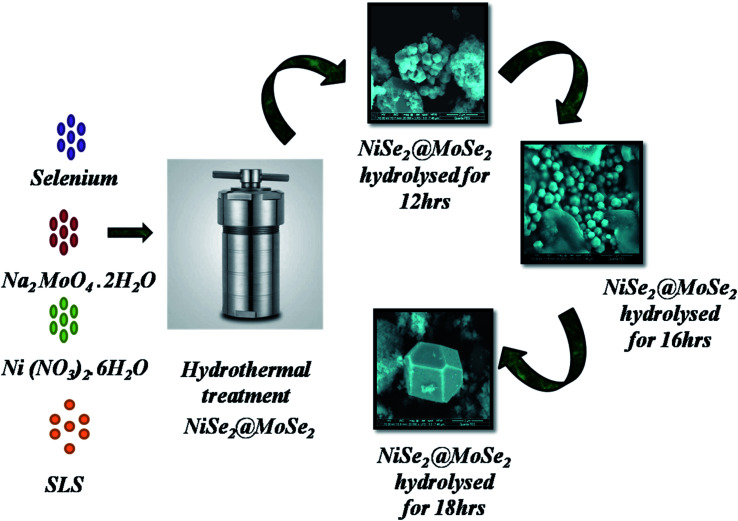
NMS hydrolyzed for 12, 16 and 18 h.

In the proposed method, the procedure used for the synthesis of NMS was explored by varying the duration of hydrolysis between 12, 16 and 18 h. 0.1 M of Ni(No_3_)_2_·6H_2_O and 0.1 M of Na_2_MoO_4_·2H_2_O were suspended in 50 ml de-ionized water and stirred for 20 min. The mixture turns black immediately after the addition of 0.2 M of selenium powder and then the surfactant sodium lauryl sulfate (SLS) is added to the prepared solution. The mixture was stirred continuously for 1 h in an autoclave, and hydrolyzed at 180 °C for 12 h, the product was referred to as NMS hydrolyzed for 12 h. After that, it cooled naturally and the obtained NMS was cleaned. The same protocol was repeated and NMS was synthesized by varying the duration, hydrolysis durations of 16 and 18 h were used to tune the composition and the structure of the as prepared samples, referred to as NMS hydrolyzed for 16 and 18 h, respectively. The synthetic procedure is schematically illustrated in Fig. 1. For preparation of the electrode, the nickel foam was first prepared by consecutive sonication using 1 M NaOH for 15–20 min and then subsequently washed with 6 M HCl. The electrode slurry, activated carbon (AC), and PVDF in 80 : 15 : 5 wt% was drop-cast onto the nickel foam and dried at 80 °C. Platinum was used as a counter electrode, Ag/AgCl as a reference electrode and 1 M aqueous KOH as an electrolyte. After conducting the experiment using the three electrodes, the asymmetric supercapacitor device was designed and fabricated by assembling the NMS (hydrolyzed for 18 h) and AC as the positive and negative electrodes in 1 M KOH using biological SP150 apparatus.

## 3. Results and discussion

**Fig. 2:**
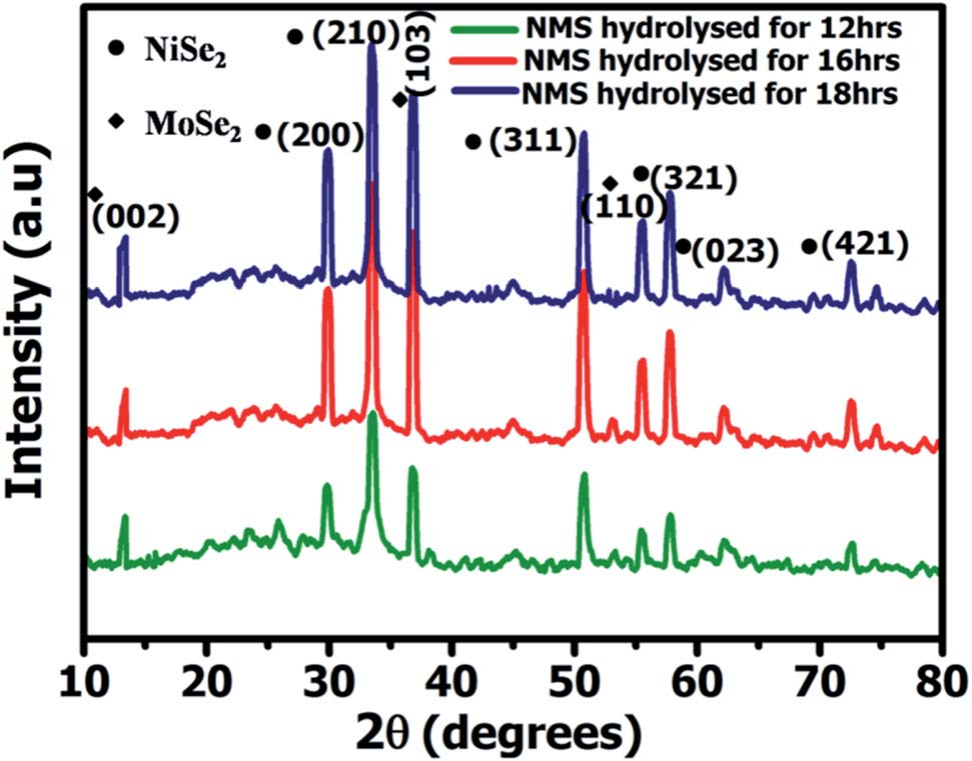
XRD spectrum of NMS (hydrolyzed for 12, 16, and 18 h) *via* hydrothermal method.

Fig. 2 shows the powder X-ray diffractometry (XRD) spectrum of the NMS samples prepared for 12, 16 and 18 h, respectively, with 2*θ* values of NiSe_2_ at 29.9°, 33.5°, 50.7°, 57.8°, 62.2°, and 72.5° corresponding to the (200), (210), (311), (321), (023) and (421) planes and the crystalline peaks of MoSe_2_ at 13.6°, 37.8°, 55.8° ascribed to the diffraction planes of (002), (110), and (103). The diffraction peaks of NiSe_2_ and MoSe_2_ were in agreement with JCPDS card numbers #89-7161 and #77-1715. Furthermore, no other impurity peaks were observed, indicating the structural purity of the NMS. The unique polyhedron nanostructure of the NMS exhibits a high crystallinity, which contributes to optimization of the high electrochemical performance.

**Fig. 3:**
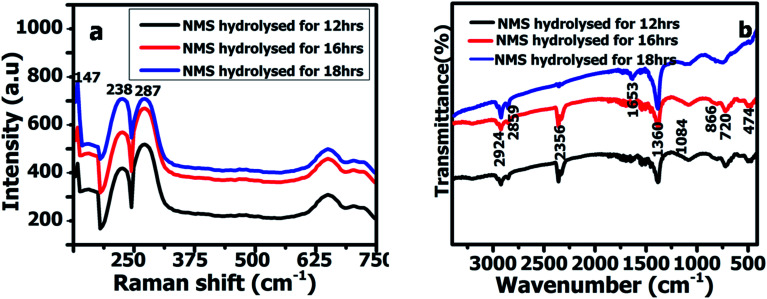
(a) FTIR spectra of NMS hydrolyzed for 12, 16 and 18 h. (b) Raman spectra of NMS hydrolyzed for 12, 16 and 18 h.

Fourier transform infrared spectroscopy (FTIR) spectra were acquired in the wave number region between 3400 and 500 cm^−1^. Fig. 3a shows the FTIR spectra for the ternary NMS nanoparticles hydrolyzed for 12, 16 and 18 h, respectively. The appearance of the typical infrared peak at 2924 and 2859 cm^−1^ is attributed to the CH_2_ vibration. The absorbance peak at 1084 cm^−1^ could be attributed to the O–H bending vibrations. The band observed at 866 cm^−1^ revealed the presence of the asymmetric stretching vibration (*ν*_as_) of O–Mo–O.^20^ The band at 720 cm^−1^ was used to explore the complex, confirming the metal oxygen vibration bonding with Se. The band at 474 cm^−1^ revealed the symmetric stretching vibration (*ν*_s_) of Se–O–Se bonding with NMS.^21^ The laser Raman spectrum was used to investigate the vibrational and rotational modes of the polyhedron structured _NMS_ nanoparticles synthesized *via* the hydrothermal route and hydrolyzed at 12, 16 and 18 h. The polyhedron structured NMS shown in Fig. 3b reveals the existence of two bands at 238 cm^−1^ and 287 cm^−1^, respectively. The band at 238 cm^−1^ revealed the A_1g_ mode and the band at 287 cm^−1^ was used to explore the E^1^_2g_ mode.^22,23^ Hence, the peaks appeared at 147 and 238 cm^−1^, corresponding to the configuration of the elemental selenium Raman curves of NiSe_2_.^24^

**Fig. 4:**
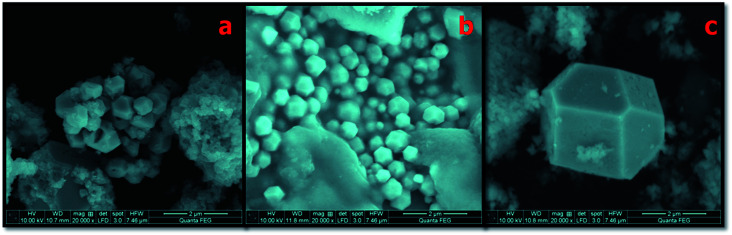
(a)–(c) Surface morphology of the NMS hydrolyzed for 12, 16 and 18 h.

The surface morphological analysis of the NMS hydrolyzed for 12, 16 and 18 h synthesized using the hydrothermal method was investigated using scanning electron microscopy (SEM), as shown in Fig. 4a–c. When NMS was hydrolyzed for 12 h, a well adhered tightly packed polyhedron coral-like surface morphology was observed, as shown in Fig. 4a. In addition, the NMS hydrolyzed for 16 h shows a sponge like polyhedron structure with a uniform distribution of the nanoparticles, which is considered to be a further suitable path for ionic transportation which enriches the high-rate capacitance compared to the NMS hydrolyzed for 12 h, as shown in Fig. 4b. Again, when NMS was hydrolyzed for 18 h, it limits the agglomeration of the prepared nanocomposites, and the efficient interfacial charge transportation is shown in Fig. 4c. The unique polyhedron nanostructure exhibits an effectively reduced diffusion resistance, which contributes to the optimization of the high electrochemical performance.^25^ On the other hand, the NMS hydrolyzed for 18 h provides a high-rate capability and an admirably stable cycle life, as a result a high performance supercapacitor is realized.

**Fig. 5:**
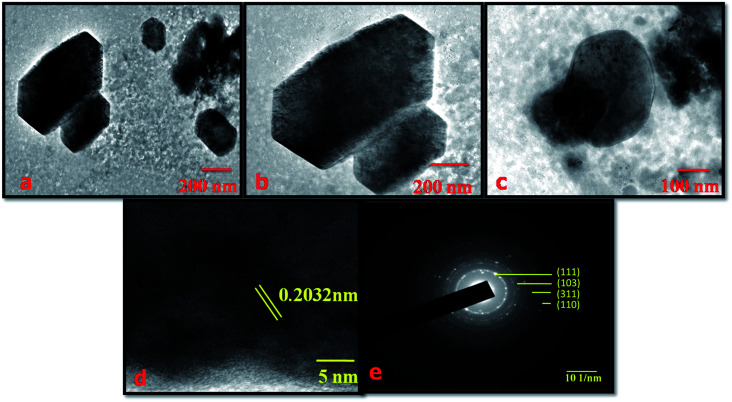
(a)–(c) TEM images of NMS hydrolyzed for 18 h, (d) fringe pattern, and (e) the SAED pattern.

The morphology of the NMS hydrolyzed for 18 h was examined using high resolution transmission electron microscopy (HR-TEM). Fig. 5a–c explored the HR-TEM image of the polyhedron structured NMS hydrolyzed for 18 h. The image displays the fine distribution of the unique polyhedron structure of the NMS hydrolyzed for 18 h. The polyhedron structure reveals that the increased particle width results in an enhancement in the ease of accessibility for diffusion of the ions through the electrolytic medium. Fig. 5d and e represent the fringe and selected area electron diffraction (SAED) pattern of the NMS hydrolyzed for 18 h.

**Fig. 6:**
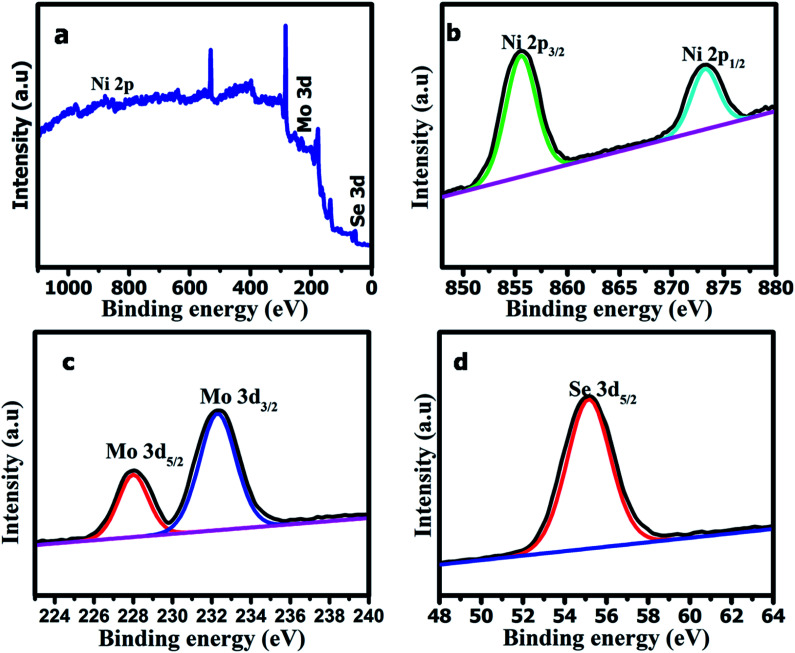
XPS survey spectra for (a) the polyhedron shaped NMS, (b) the Mo3d spectrum, (c) the Se3d spectrum, and (d) the Ni2p spectrum.

To quantify the chemical bonding and composition of the _NMS_ nanomaterials, the XPS spectra were studied and enabled detailed investigation of the oxidation state of the elements. The XPS wide scan survey spectrum of the polyhedron structured NMS reveals the existence of high resolution Ni2p, Mo3d, and Se3d as illustrated in Fig. 6a. The XPS spectra of Ni2p fitted at 855.6 and 873.2 eV revealing the presence of Ni^2+^2p_3/2_ and Ni^2+^2p_1/2_.^26^ The core level Mo3d peaks at 228.8 and 232.2 eV revealed the presence of Mo^4+^3d_5/2_ and Mo^4+^3d_3/2_ and substantiates the +4 oxidation (Fig. 6b). The peak at 55.1 eV was used to explore the metal selenide electronic state of Se3d_5/2_ (Fig. 6c). From the chemical compositional analysis it is clear that a polyhedron shaped NMS has been formed.^27^

**Fig. 7:**
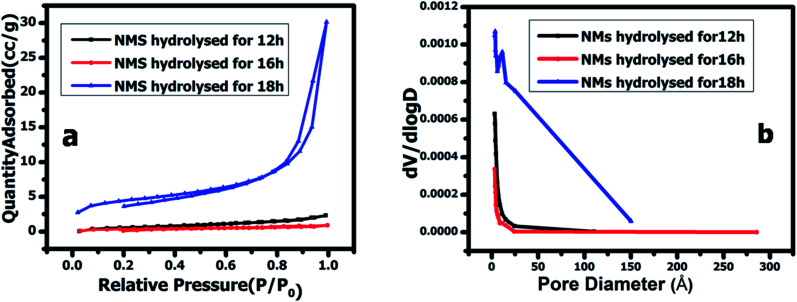
(a) N_2_ sorption isotherms, and the (b) pore size distribution of NMS hydrolyzed for 12, 16, and 18 h.

The Brunauer–Emmett–Teller (BET) analysis revealed the specific surface area and pore size of the as-prepared NMS hydrolyzed for 12, 16 and 18 h, respectively. The N_2_ sorption isotherm of the as prepared NMS shows similar type V isotherms, as shown in Fig. 7a. Furthermore, the NMS hydrolyzed for 18 h shows an enhanced N_2_ adsorption compared to the NMS hydrolyzed for 12 and 16 h. The Barrett–Joyner–Halenda method was used to calculate the distribution of the pore diameter and is depicted in Fig. 7b. The pore sizes of the NMS hydrolyzed for 12, 16 and 18 h were found to be 3.094, 3.118, and 3.492 nm respectively, revealing their mesoporous structure and their corresponding specific surface area was found to be 0.742, 1.905, and 9.775 m^2^ g^−1^. The elevated surface area and mesoporous nature of the NMS hydrolyzed for 18 h enhances the electrochemical activity by decreasing the ion pathways, improving the contact area, enhancing the redox active sites, increasing the electrolyte penetration *via* the mesopores, and facilitating the charge transfer kinetics.

### 3.1. Electrochemical measurements

The working electrode was equipped *via* the traditional slurry coating method. To assess the practical aspects of the NMS electrode, an asymmetric device (NMS hydrolyzed for 18 h//AC) was fabricated using the NMS hydrolyzed for 18 h as a positive electrode and AC as the negative electrode in 1 M KOH employing a biological SP-150 instrument. Before the electrochemical testing of the NMS hydrolyzed for 18 h//AC asymmetric device, a CV test for both the positive and negative electrode was carried out for the three electrodes at 10 mV s^−1^. In a three-electrode system, the active material mass coated on a 1 × 1 cm^2^ Ni foam current collector was estimated as approximately 2 mg and the specific capacitance was obtained from the three-electrode system for both the active material and the AC. In the case of the two electrode system, however, it is necessary to perform a mass balance *m*^−^/*m*^+^=(*C*^+^ × *V*^+^)/(*C*^−^ × *V*^−^). After that, it was carefully optimized and coated with 2 and 6 mg for the positive and negative electrodes, respectively.

#### Polyhedron shaped NMS electrochemical properties

**Fig. 8:**
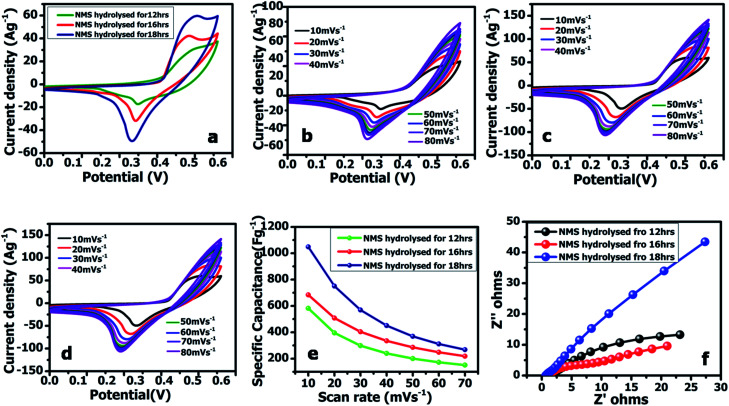
(a) Comparison of the CV curves of the active electrode hydrolyzed for 12, 16 and 18 h at 10 mV s^−1^. (b) CV curves of the active electrode NMS hydrolyzed for 12 h, (c) 16 h, and (d) 18 h. (e) Impedance spectra. (f) Specific capacitance at varying scan rates.

The electrochemical performance of the prepared polyhedron structured NMS was examined for the three electrodes for the supercapacitor *via* 1 M KOH. The comparative CV curves of the active electrode hydrolyzed for 12, 16 and 18 h at 10 mV s^−1^ is shown in Fig. 8a. Fig. 8b–d is reveals the CV curves of the active electrode at various sweep rates in an operating voltage of 0–0.6 V (*vs.* Ag/AgCl). The polyhedron structured NMS demonstrates two pairs of redox peaks, signifying a standard faradaic pseudo capacitance. It should be noted that the current area in the CV and the peak current apparently increases with the increasing scan rate and the redox peak pair are still visible at 30 mV s^−1^. This consequence indicates that the NMS hydrolyzed for 18 h demonstrates a rapid reversible redox process and a high-rate ability in aqueous KOH. The perfect shape retention of the CV curve indicates that the NMS hydrolyzed for 18 h has an enhanced electrochemical reversibility and excellent rate capability. It was found that the CV curves of the polyhedron structured active electrode NMS hydrolyzed for 18 h are similar to the NMS hydrolyzed for 12 h and the NMS hydrolyzed for 16 h, however, the NMS hydrolyzed for 18 h showed a greater electrochemical active area, representing a much higher faradaic redox behavior and large capacity.^28^ It is worth noting that the specific capacitance of the NMS hydrolyzed for 12, 16 and 18 h was enhanced to 528, 684, and 1048 F g^−1^ under 10 mV s^−1^ when the scan rate was boosted-up to 100 mV s^−1^ and the specific capacitance decreased (Fig. 8e).^29^ The NMS hydrolyzed for 18 h demonstrates an exceptional electrochemical performance.

**Fig. 9:**
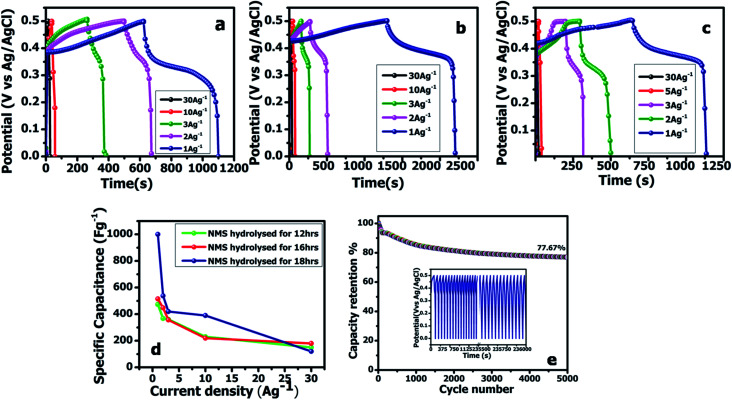
(a)–(c) Electrochemical performances: charge discharge curves of NMS hydrolyzed for 12, 16 and 18 h. (d) Gravimetric analysis of the specific capacitance of the electrode at diverse current densities. (e) Stability of the cycle life and capacity retention of NMS hydrolyzed for 18 h.

The galvanostatic charge–discharge curves were collected between 0–0.5 V (*vs.* Ag/AgCl). The equivalent discharge capacitance curves of the charge discharge are shown in Fig. 9a–c. The polyhedron structured NMS hydrolyzed for 18 h exhibits a high specific capacitance of 1000 F g^−1^ at 1 A g^−1^, higher than that for the NMS hydrolyzed for 12 h (473 F g^−1^) and NMS hydrolyzed for 16 h (515 F g^−1^). Encouragingly, the specific capacitance of the active electrode formed from NMS hydrolyzed for 18 h remains as high as 420 F g^−1^, even at 3 A g^−1^, and highlights the high capacitance of the NMS hydrolyzed for 18 h. Furthermore, the electrode also demonstrates the battery behavior and the specific capacity was calculated as 65.6, 71.5 and 138.8 mA h g^−1^ for the NMS hydrolyzed for 12, 16, and 18 h using the equation 
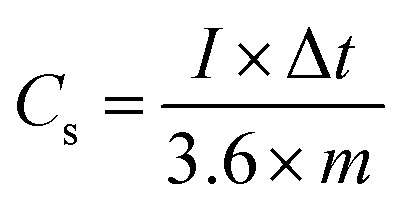
. The rate capability of the NMS hydrolyzed for 18 h electrode was examined (Fig. 8d). In the charge discharge process at a low current density the electrolyte ions penetrate into the interior electrode surface resulting in the higher *C*_s_ value. Hence, the polyhedron structure of the NMS hydrolyzed for 18 h shows the high specific capacitance, which is favorable for practical outcomes.

Electrochemical impedance spectroscopy (EIS) was used to scrutinize the reaction kinematics and electrical conductivity of the active electrodes of NMS hydrolyzed for 12, 16 and 18 h I and are reported in Fig. 8f. The electrodes made from NMS hydrolyzed for 12, 16 and 18 h demonstrated the following resistivity values: 1.84, 1.38 and 0.66 Ω. Therefore, the polyhedron structure NMS hydrolysed for 18 h showed the low solution resistance *i.e.*, *R*_s_ = 0.66 Ω in the low frequency region reveals it highest conductivity compared to other samples. ^30^ The results of the lower equivalent series resistance indicate that the polyhedron shaped NMS hydrolyzed for 18 h is more favorable as an active material in contact with the electrolyte.^31^ The high conductivity may be a factor that determines the high-rate capacitance.

The cycling performance is also another vital factor used to explore the service life of the supercapacitor electrode material. Fig. 9e reveals the cycling performance of the active electrode NMS hydrolyzed for 18 h according to the charge discharge analysis at 10 A g^−1^ for 5000 cycles. The cycle stability of the active electrode exhibits a capacitive retention of 77.67% owing to the presence of selenium, which improves the intrinsic surface area and enhances the ionic diffusion. Furthermore, the unique polyhedron structure accommodates the variation in volume variation and the charge/discharging guarantees the structural stability and cycle life.^32^ The attractive stability performance of the active electrode hydrolyzed for 18 h is attributed to its unique polyhedron structures, suggesting that this sort of surface morphology is suitable for use as a supercapacitor active electrode.^33^

To further study the electrochemical capability of the NMS electrode, an asymmetric supercapacitor was constructed using the NMS hydrolyzed for 18 h as a positive and AC as a negative electrode in 1 M KOH. The CV curves for the NMS hydrolyzed for 18 h//AC at a diverse scan rate shows a pair of redox peaks similar to the three electrodes. The two electrodes are balanced using the mass balance method,1
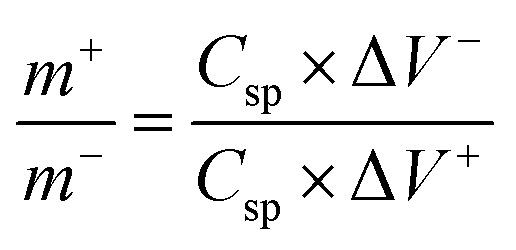


**Fig. 10:**
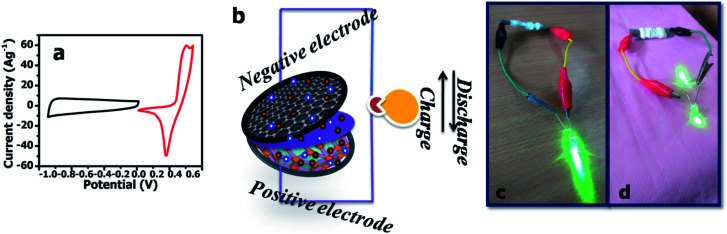
(a) CV profile of the active electrode NMS hydrolyzed for 18 h and AC using 1 M KOH as an electrolyte at a scan rate of 10 mV s^−1^. (b) Schematic representation of the asymmetric device. (c) and (d) LED lit up using the NMS hydrolyzed for 18 h//AC.

**Fig. 11:**
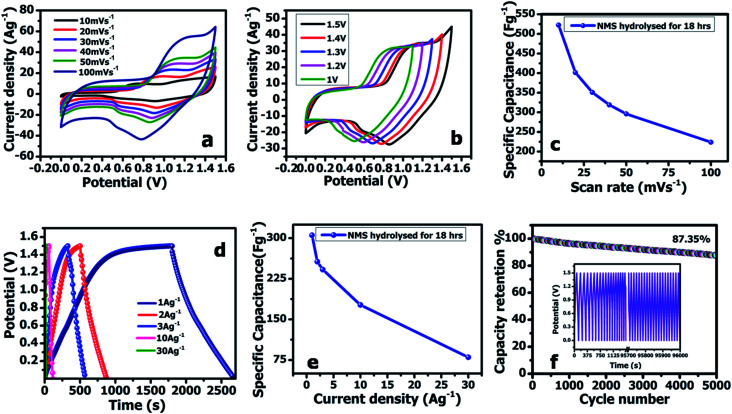
(a) Measured CV curve of NMS hydrolyzed for 18 h. (b) CV curve measured at different potential windows (NMS hydrolyzed for 18 h//AC). (c) Specific capacitance at various scan rates. (d) Galvanostatic charge/discharge curves of the asymmetric device. (e) Current density dependence and specific capacitance. (f) Capacity retention of the fabricated asymmetric device at a current density of 10 A g^−1^.

The two electrodes possess different potential windows of NMS hydrolyzed for 18 h (0 to 0.6 V) and AC (−1 to 0 V) (Fig. 10a). Accordingly, the voltage of the NMS hydrolyzed for 18 h//AC asymmetric supercapacitor reached 1.5 V. When the potential reaches 0–1.5 V, the NMS hydrolyzed for 18 h//AC device works well, hence the polarization achieved at 0–1.5 V indicating the proper working potential window of the asymmetric supercapacitor should be 0–1.5 V. Consequently, the asymmetric supercapacitor possesses an ideal capacitive behavior and good reversibility, as shown in Fig. 11a. Fig. 11b shows the CV curves of NMS at various potential windows from 1.1–1.5 V. From the charge–discharge curves, the specific capacitance achieved using the NMS hydrolyzed for 18 h//AC is shown in Fig. 11d according to eqn (2). The calculated values were 305, 256, 242, 176 and 80 F g^−1^ at 1, 2, 3, 10 and 30 A g^−1^. The decrease in the capacitance helps increase the current density (Fig. 11e). The progress of the cycle life is an important criteria of the prepared active electrode NMS hydrolyzed for 18 h. The cycle stability was measured at 10 A g^−1^ and 87, and a 35% capacity retention is attained for 5000 continuous cycles, which demonstrates the good cycle life of the active electrode material, as shown in Fig. 11f.2
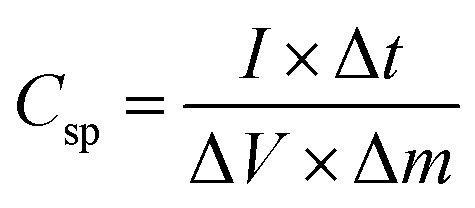


The properties of the asymmetric supercapacitor can enhance their energy density. A high energy density under the premise of a high-power density is a major constraint for good supercapacitors. The NMS//AC asymmetric device exhibited a maximum power density and the resultant energy density is well reported, as shown in Table 1. A schematic diagram of the asymmetric device is shown in Fig. 10b. In order to validate the special practical applicability, the asymmetric device was fabricated to effectively light a green light emitting diode (LED), as shown in Fig. 10c and d. The power density (P) and specific energy density (*E*) were calculated^34^ as follows:3
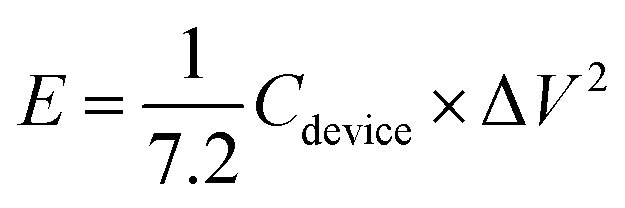
4
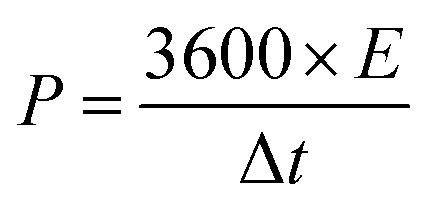


**Table 1:** Prepared material values

Potential window (V)	Energy density (W h kg^−1^)	Power density (W kg^−1^)
1.5	94	369
	79	738
	75	1115
	54	3667
	24	10 800

## 4. Conclusions

In summary, a novel polyhedron structured NMS was successfully synthesized. The unique finely distributed NMS was hydrolyzed for 18 h, and was observed to possess a fine crystalline nature and deliver a high capacitance of 1000 F g^−1^ (138.8 mA h g^−1^) achieved at 1 A g^−1^. Furthermore, the active electrode NMS hydrolyzed for 18 h exhibited cyclic stability with a retention of 77.67% for 5000 continuous cycles, indicating its excellent electrochemical performance and cyclic stability. In addition, as an asymmetric device, the electrode exhibited an outstanding electrochemical performance and an improved specific capacitance of 305 F g^−1^ that can be achieved at 1 A g^−1^, together with a good cycle life with 87.35% capacity retention over 5000 cycles. Finally, the potential applicability of the NMS hydrolyzed for 18 h//AC device was also verified using an energizing LED. The results obtained for the NMS electrode indicate that it could serve as a promising candidate for supercapacitor applications.

## Conflicts of interest

There are no conflicts to declare.

## Supplementary Material
